# Charcot Spinal Arthropathy-Induced Progression From Upper to Lower Motor Neuron Bowel Syndrome

**DOI:** 10.7759/cureus.15073

**Published:** 2021-05-17

**Authors:** Danyon J Anderson, Nathan Li, Hefei Liu, Trenton Reinicke, Christopher White

**Affiliations:** 1 School of Medicine, Medical College of Wisconsin, Wauwatosa, USA; 2 Clinical and Translational Epidemiology Unit, Massachusetts General Hospital, Boston, USA; 3 Physical Medicine and Rehabilitation, Medical College of Wisconsin, Wauwatosa, USA

**Keywords:** charcot spinal arthropathy, spinal cord injury, neurogenic bowel, upper motor neuron bowel, lower motor neuron bowel

## Abstract

Charcot spinal arthropathy is a progressively degenerative joint disorder of the vertebrae. Historically, it was a common consequence of tertiary syphilis. Currently, it is a rare complication of spinal cord injury (SCI). We present the case of a 28-year-old patient with paraplegia who developed progressive, neurogenic bowel dysfunction due to Charcot spinal arthropathy. Our patient had upper motor neuron bowel syndrome secondary to SCI which advanced to lower motor neuron bowel syndrome. Charcot spinal arthropathy should be considered as a possible cause for symptom progression in SCI patients. This case illustrates the connection between Charcot spine and lower motor neuron dysfunction in the setting of prior upper motor neuron dysfunction.

## Introduction

Traumatic spinal cord injury (SCI) may cause motor paralysis and sensory changes due to spinal cord damage, ischemia, peripheral inflammatory cell infiltration, and proapoptotic signaling [1]. SCI can cause dysfunction of upper motor neurons innervating structures distal to the lesion. Neurogenic bowel, more specifically, upper motor neuron bowel syndrome, is a potential consequence of SCI-induced upper motor neuron dysfunction [2,3].

Charcot spinal arthropathy is a degenerative joint disease of the spine and can be secondary to SCI [4]. After vertebral integrity is compromised by the initial SCI, repeated microtrauma may occur as damaged cartilage, ligaments, and bone progressively compress the spine [5]. This repeated trauma, which is called Charcot spinal arthropathy, may cause spinal compression and concomitant symptom progression after the initial SCI.

Though Charcot spinal arthropathy may arise secondary to SCI, it is a distinct entity. Therefore, different anatomic features may be affected in Charcot spinal arthropathy than in SCI. Here, we present a case of progressive, total motor neuron bowel dysfunction secondary to Charcot spinal arthropathy.

## Case presentation

**Figure 1:**
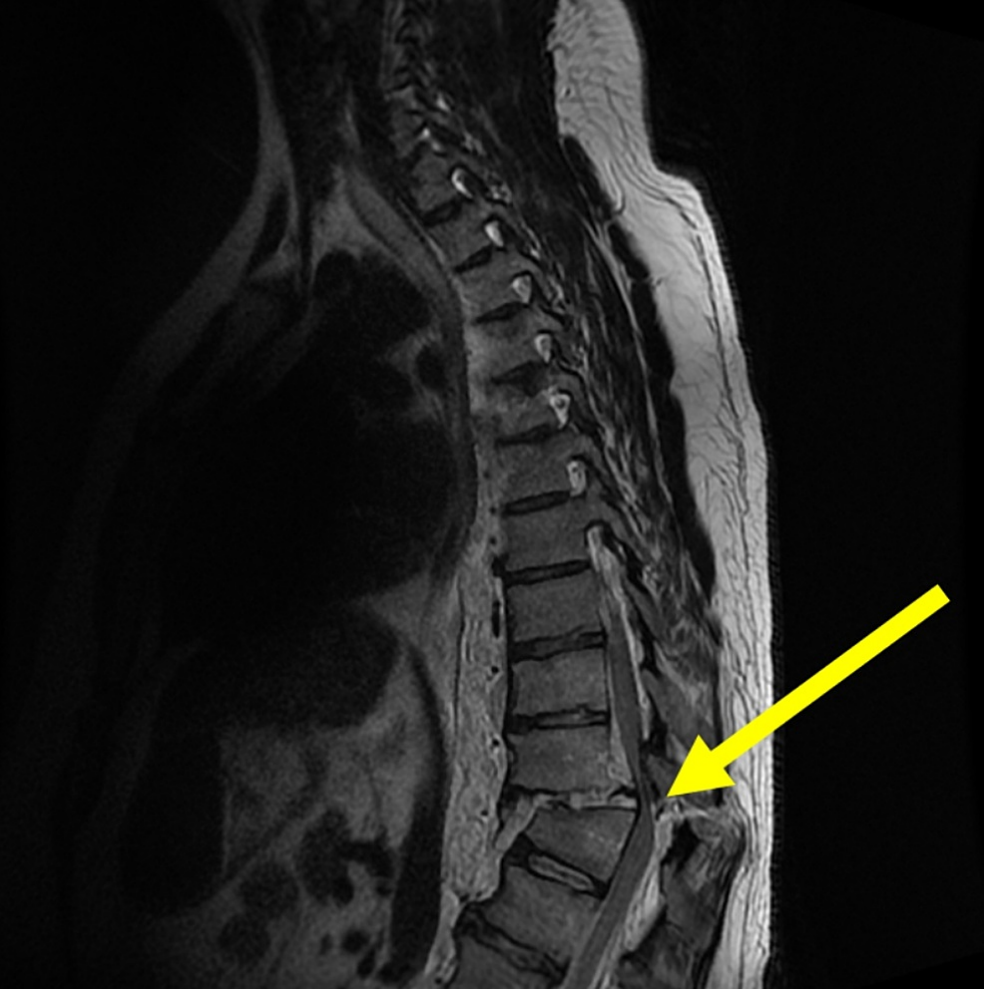
Initial injury. Acute T11 burst fracture deformity, associated with marked retropulsion of the T11 middle column into the spinal canal; ventral epidural hematoma; severe spinal cord stenosis and cord compression with cord edema; and bilateral T11-12 jumped facets. Suspected complete disruption of the anterior longitudinal ligament, posterior longitudinal ligament, interspinous ligament, ligament flavum, and facet joint capsules at the T10-11 level.

**Figure 2:**
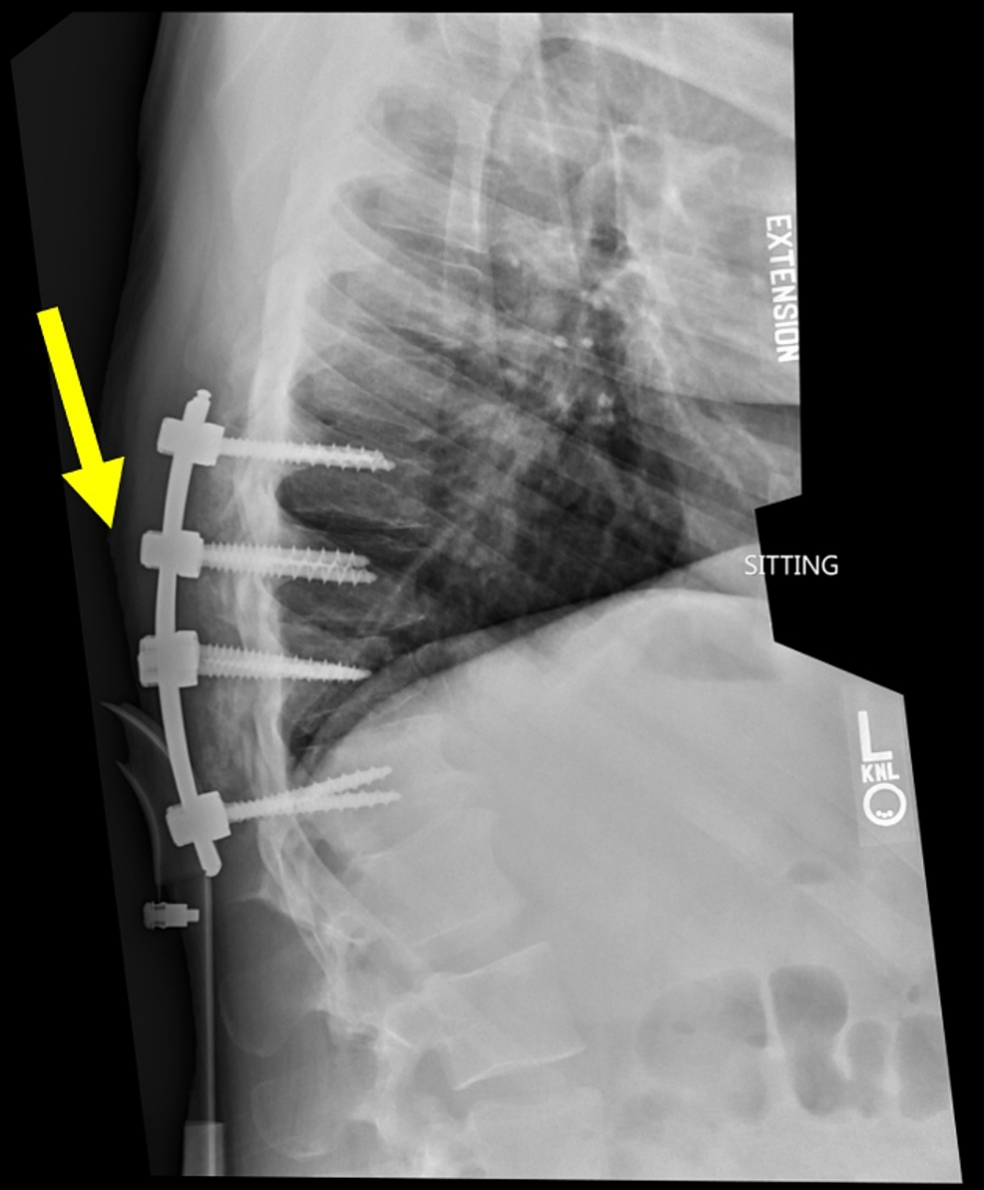
Post-surgery. T9-T12 posterior spinal fusion without hardware complication or dynamic instability.

A 28-year-old male presented to an academic medical center after a fall from a 10-foot-tall ladder with loss of consciousness. Magnetic resonance imaging (MRI) showed T10-T11 fracture subluxation resulting in SCI (Figure 1). Open reduction and internal fixation of fracture subluxation were performed (including T10 and T11 decompressive laminectomies and fracture reduction). An arthrodesis (posterolateral fusion) was performed among T9-T10, T10-T11, and T11-T12 using a morselized autograft (Figure 2).

**Figure 3:**
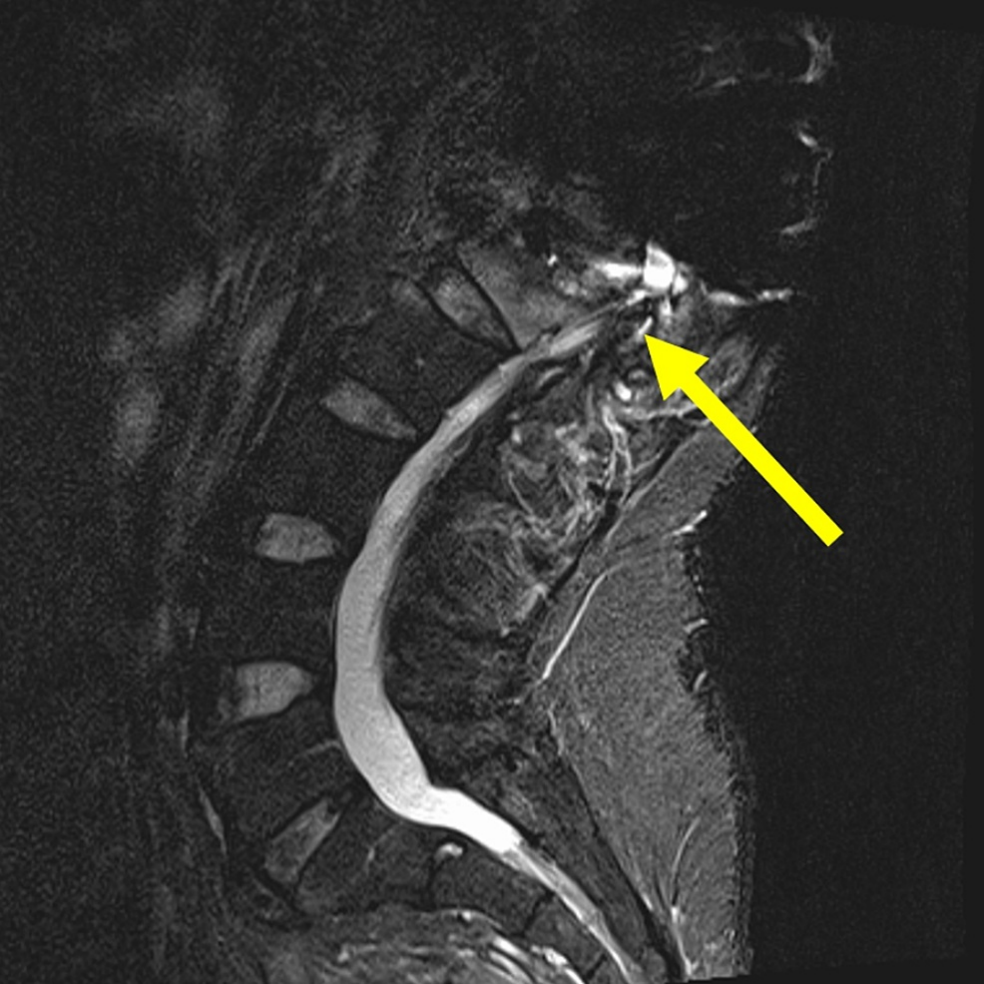
Charcot spinal arthropathy. Severe narrowing with compression of the spinal cord/conus medullaris at this level.

As a result of his injury, he was diagnosed with a T7 American Spinal Injury Association Impairment Scale Class A (complete) paraplegia with lower extremity paralysis and loss of sensation, neurogenic bladder, neurogenic erectile dysfunction, muscle spasticity, and neurogenic bowel. The patient’s neurogenic bowel was initially due to T10-T11 upper motor neuron damage from his SCI. His constipation and diarrhea were well controlled with a standard upper motor neuron bowel program consisting of daily digital stimulation, a bisacodyl suppository, stool softeners as needed, and avoidance of gas promoting agents. After two years, his symptoms progressed to increased constipation and inconsistent emptying. As the course of his neurogenic bowel progressed, his upper motor neuron program decreased in effectiveness. Rectal examination revealed no tone to his external/internal anal sphincter, consistent with lower motor neuron dysfunction. An MRI of his lower spine showed degenerative changes with spinal cord compression of the conus medullaris consistent with Charcot spinal arthropathy. This likely explains his changes in symptoms and bowel management (Figure 3). A lower motor neuron bowel program was started, including docusate sodium, senna, magnesium hydroxide, bisacodyl, more dietary restrictions, and more time to defecate. At the time of writing, the patient was considering a colostomy due to disease progression and is a candidate for circumferential fusion and osteosynthesis.

## Discussion

Charcot spinal arthropathy is a degenerative joint condition that arises when there is impaired afferent innervation [6]. Impaired afferent innervation results in decreased proprioception and deep pain sensation. Because proprioception and pain can be protective against movements and postures that cause spinal cord damage, Charcot spinal arthropathy is associated with compressive neuropathy of the spine.

Charcot spinal arthropathy was historically associated with tertiary syphilis; however, it is now more commonly seen as a complication of SCI in the antibiotics era [7]. Charcot spinal arthropathy from syphilis has been cited to be a cause of neurogenic bowel [8-10]. One case of neurogenic bowel secondary to non-syphillis-associated Charcot spinal arthropathy has been published [11]. The connection between Charcot spinal arthropathy and worsening neurogenic bowel in the setting of SCI has not been well explored. Indeed, this is the first reported progression of upper to lower motor neuron bowel syndrome caused by Charcot spinal arthropathy.

We present a case of neurogenic bowel secondary to SCI that was complicated by Charcot spinal arthropathy. This case illustrates that Charcot spinal arthropathy is a possible cause for progressing neurogenic bowel symptoms after SCI. Recognizing this progression from upper to lower motor neuron bowel syndrome can guide appropriate treatment. Upper motor neuron and lower motor neuron bowel programs are distinct. Lower motor neuron neurogenic bowel treatment requires significantly more oral medications, more time for defecation, and more frequent defecations. Treatment of upper motor neuron neurogenic bowel requires, on average, 0.46 defecations per day, 185 minutes for defecation per week, and 0.27 oral medications for bowel care [12]. Treatment of lower motor neuron neurogenic bowel requires, on average, 1.95 defecations per day, 396 minutes for defecation per week, and 0.95 oral medications for bowel care [12]. When progression from upper to lower motor neuron bowel syndrome is diagnosed, patients should be prescribed additional oral medications (such as senna) and their bowel routine should be changed to allow for more frequent defecations of longer duration. Furthermore, Charcot spinal arthropathy can be treated surgically with circumferential fusion and osteosynthesis [13]. It is important to consider Charcot spine on the differential for progressing neurogenic bowel secondary to SCI.

## Conclusions

Charcot spinal arthropathy can cause progression from upper motor neuron neurogenic bowel to lower motor neuron neurogenic bowel in SCI patients. Recognition of Charcot spinal arthropathy in this setting guides bowel programs and can be an indication for surgery.
